# Community-Based Testing Sites for SARS-CoV-2 — United States, March 2020–November 2021

**DOI:** 10.15585/mmwr.mm7049a3

**Published:** 2021-12-10

**Authors:** Mark F. Miller, Min Shi, Alison Motsinger-Reif, Clarice R. Weinberg, Joseph D. Miller, Erin Nichols

**Affiliations:** ^1^National Institute of Environmental Health Sciences, Research Triangle Park, North Carolina; ^2^U.S. Public Health Service; ^3^National Center for Emerging and Zoonotic Infectious Diseases, CDC; ^4^CDC COVID-19 Response Team; ^5^National Center for Health Statistics, CDC.

Immediately following the March 13, 2020 declaration of COVID-19 as a national emergency (*1*), the U.S. government began implementing national testing programs for epidemiologic surveillance, monitoring of frontline workers and populations at higher risk for acquiring COVID-19, and identifying and allocating limited testing resources. Effective testing supports identification of COVID-19 cases; facilitates isolation, quarantine, and timely treatment measures that limit the spread of SARS-CoV-2 (the virus that causes COVID-19); and guides public health officials about the incidence of COVID-19 in a community. A White House Joint Task Force, co-led by the Department of Health and Human Services (HHS) and the Federal Emergency Management Agency (FEMA), created the Community-Based Testing Sites (CBTS) program working with state and local partners (*2*). This report describes the timeline, services delivered, and scope of the CBTS program. During March 19, 2020–April 11, 2021, the CBTS program conducted 11,661,923 SARS-CoV-2 tests at 8,319 locations across the United States and its territories, including 402,223 (3.5%) administered through Drive-Through Testing, 10,129,142 (86.9%) through Pharmacies+ Testing, and 1,130,558 (9.7%) through Surge Testing programs. Tests administered through the CBTS program yielded 1,176,959 (10.1%) positive results for SARS-CoV-2. Among tested persons with available race data,* positive test results were highest among American Indian or Alaska Native (14.1%) and Black persons (10.4%) and lowest among White persons (9.9%), Asian persons (7.3%), and Native Hawaiian or Other Pacific Islanders (6.4%). Among persons with reported ethnicity, 25.3% were Hispanic, 15.9% of whom received a positive test result. Overall, 82.0% of test results were returned within 2 days, but the percentage of test results returned within 2 days was as low as 40.7% in July 2020 and 59.3% in December 2020 during peak testing periods. Strong partnerships enabled a rapid coordinated response to establish the federally supported CBTS program to improve access to no-charge diagnostic testing, including for frontline workers, symptomatic persons and close contacts, and persons living in high-prevalence areas. In April 2021, the CBTS Pharmacies+ Testing and Surge Testing programs were expanded into the Increasing Community Access to Testing (ICATT) program. As of November 12, 2021, the CBTS and ICATT programs conducted approximately 26.6 million tests with approximately 10,000 active testing sites. Although the CBTS program represented a relatively small portion of overall U.S. SARS-CoV-2 testing, with its successful partnerships and adaptability, the CBTS program serves as a model to guide current community-based screening, surveillance, and disease control programs, and responses to future public health emergencies.

The CBTS program was created by a White House Joint Task Force, co-led by HHS and FEMA in March 2020 (*1*). The program comprised three distinct efforts to provide federally funded, no-charge testing: 1) Drive-Through Testing*,* in collaboration with state and local partners; 2) Pharmacies+ Testing, through a federal government collaboration with commercial partners, including retail pharmacies and other contract service providers; and 3) Surge Testing, for rapid surveillance of at-risk communities through increased testing capacity in support of state, tribal, local, and territorial health agencies. Individual testing sites provided predominantly nucleic acid amplification tests and were established with varying dates and durations of operation to meet the needs of the specific communities served.

Within 72 hours of its initiation on March 13, 2020, the CBTS Drive-Through Testing program developed a concept of operations for federally supported, state-managed, and locally executed testing facilities (*2*). Ultimately, 39 sites provided low transmission–risk testing, increased the availability of local resources, and provided access for at-risk populations.^†^ State and local agencies provided facilities, staffing, public communications, and operational management. The federal government provided a Chief Medical Officer under whose medical license all SARS-CoV-2 medical testing was ordered and reported. In addition, the federal government provided supportive staffing in the form of U.S. Public Health Service officers with medical expertise, additional operational management and logistical distribution of testing supplies, and personal protective equipment. The federal government also contracted with the private sector to provide services, such as specimen transport, sample analysis, and communication of results. Positive test results were reported to state and local health departments for follow-up contact tracing and local support services. Specimen collection began on March 19, 2020, and continued until operations were transferred to the state or until other local testing programs met community demand and the site was closed; all 39 locations were closed or transitioned to state and local programs by July 31, 2020.

With projections that substantial testing would be needed to track and control the spread of COVID-19, an expanded CBTS Pharmacies+ Testing program was launched on April 5, 2020, establishing partnerships with retail pharmacies and other providers leveraging their expansive networks to increase community-level testing access. Testing was provided at 7,708 locations nationwide at sites supported through HHS contracts and operated through collaborations between pharmacies and analytical laboratories. As the pandemic progressed, the CBTS Surge Testing program was established on July 7, 2020 and, through April 11, 2021, provided increased testing capacity in 658 communities where a sharp increase in COVID-19 incidence was occurring or predicted.

The number of testing locations, tests administered, and results (positive, negative, and indeterminate) were assessed for the Drive-Through Testing, Pharmacies+ Testing, and Surge Testing programs. The age, race and ethnicity, and symptom status of persons tested through these programs was also assessed. Because of variations in reporting across states, aggregate data on these variables were unavailable for persons tested in the CBTS Drive-Through Testing program; thus, these data were not included in analyses. Data for this analysis came from COVIDResponder,^§^ a data platform supported by FEMA and HHS. This platform provided an interface for testing sites to submit results and a secure central data repository for site-level and aggregate data, site reports, and supply tracking, including interactive dashboards, to inform ongoing response decisions. Statistical testing was not performed because of the large number of tests conducted, which could result in statistically significant differences in the absence of clinical significance. This activity was reviewed by CDC and conducted consistent with applicable federal law and CDC policy.^¶^

During March 19, 2020–April 11, 2021, the CBTS program conducted 11,661,923 SARS-CoV-2 tests at 8,319 locations across the United States and its territories. The program included 402,223 (3.5%) tests administered through Drive-Through Testing, 10,129,142 (86.9%) through Pharmacies+ Testing, and 1,130,558 (9.7%) through Surge Testing. Tests administered through all CBTS programs yielded 1,176,959 (10.1%) positive results, 10,430,749 (89.4%) negative results, and 54,215 (0.5%) indeterminate results, including 59,195 (14.7%) positive results, 337,255 (83.9%) negative results, and 5,773 (1.4%) indeterminate results from the CBTS Drive-Through Testing program.

Among persons tested through the Pharmacies+ Testing and Surge Testing programs, 67.8% were adults aged 20–54 years, and 42.3% were symptomatic (Table 1). Among 9,396,284 (83.5%) tested persons for whom race was reported, 54.3% were White persons (9.9% of whom received positive test results), 11.6% were Black persons (10.4% positive), 6.6% were Asian persons (7.3% positive), 0.5% were American Indian or Alaska Native persons (14.1% positive), 0.9% were Native Hawaiian or Other Pacific Islanders (6.4% positive), and 27.5% were other races (9.8% positive). Among 6,121,887 (54.4%) tested persons with reported ethnicity, 25.3% were Hispanic, 15.9% of whom received a positive test result. Overall, the highest percentage of positive test results was among persons aged <20 years and 45–54 years (10.7%) and among persons aged ≥85 years (11.5%). The percentage of positive test results was higher among males (10.8%) than among females (9.2%).

**TABLE 1:** Demographic characteristics of persons receiving SARS-CoV-2 testing, by positive test result and symptom status — Community-Based Testing Sites program, United States, March 2020–September 2021

Characteristic	Pharmacies+ Testing sites			Surge Testing sites			Combined sites		
	No. (%)*	No./total no. (%)		No. (%)*	No./total no. (%)		No. (%)*	No./total no. (%)	
		Positive test results^†^	Symptomatic^§^		Positive test results^†^	Symptomatic^§^		Positive test results^†^	Symptomatic^§^
**Total**	**10,129,142** **(100)**	**1,039,495/10,084,450** **(10.3)**	**3,441,713/7,857,366** **(43.8)**	**1,130,558** **(100)**	**78,269/1,126,808** **(6.9)**	**304,316/1,006,749** **(30.2)**	**11,259,700** **(100)**	**1,117,764/11,211,258** **(10.0)**	**3,746,029/8,864,115** **(42.3)**
**Race,^¶^ irrespective of ethnicity**									
White	4,394,142 (43.4)	452,277/4,382,208 (10.3)	1,722,676/3,829,514 (45.0)	710,707 (62.9)	49,503/709,025 (7.0)	208,484/646,484 (32.3)	5,104,849 (45.3)	501,780/5,091,233 (9.9)	1,931,160/4,475,998 (43.1)
AI/AN	49,030 (0.5)	6,880/48,838 (14.1)	21,807/42,858 (50.9)	0 (—)	0 (—)	0 (—)	49,030 (0.4)	6,880/48,838 (14.1)	21,807/42,858 (50.9)
Asian	534,095 (5.3)	42,426/532,584 (8.0)	175,596/451,399 (39.0)	86,885 (7.7)	2,702/86,725 (3.1)	16,035/83,945 (19.1)	620,980 (5.5)	45,128/619,309 (7.3)	191,631/535,344 (35.8)
Black	959,567 (9.5)	105,435/956,309 (11.1)	348,210/780,820 (44.6)	136,348 (12.1)	7,620/135,732 (5.6)	29,904/106,511 (28.1)	1,095,915 (9.7)	113,055/1,092,041 (10.4)	378,114/887,331 (42.6)
NH/OPI	63,209 (0.6)	4,947/63,042 (7.9)	19,790/57,483 (34.4)	19,748 (1.8)	380/19,741 (1.9)	2,936/19,468 (15.1)	82,957 (0.7)	5,327/82,783 (6.4)	22,726/76,951 (29.5)
Other	2,345,069 (23.2)	226,120/2,324,965 (9.7)	801,445/1,835,589 (43.7)	97,484 (8.6)	11,068/96,808 (11.4)	30,126/81,923 (36.8)	2,442,553 (21.7)	237,188/2,421,773 (9.8)	831,571/1,917,512 (43.4)
NR	1,784,030 (17.6)	201,410/1,776,504 (11.3)	352,189/859,703 (41.0)	79,386 (7.0)	6,996/78,777 (8.9)	16,831/68,418 (24.6)	1,863,416 (16.6)	208,406/1,855,281 (11.2)	369,020/928,121 (39.8)
**Ethnicity,^¶ ^irrespective of race**									
Hispanic	1,325,263 (13.1)	217,404/1,319,638 (16.5)	508,835/1,013,936 (50.2)	223,335 (19.8)	28,059/221,348 (12.7)	69,280/176,940 (39.2)	1,548,598 (13.8)	245,463/1,540,986 (15.9)	578,115/1,190,876 (48.6)
Non-Hispanic	3,991,221 (39.4)	394,131/3,979,848 (9.9)	1,516,108/3,425,792 (44.3)	582,068 (51.5)	31,998/581,008 (5.5)	158,274/536,526 (29.5)	4,573,289 (40.6)	426,129/4,560,856 (9.3)	1,674,382/3,962,318 (42.3)
NR	4,812,658 (47.5)	427,960/4,784,964 (8.9)	1,416,770/3,417,638 (41.5)	325,155 (28.8)	18,212/324,452 (5.6)	76,762/293,283 (26.17)	5,137,813 (45.6)	446,172/5,109,416 (8.7)	1,493,532/3,710,921 (40.3)
**Age group, yrs**									
<20	1,039,254 (10.3)	117,084/1,034,942 (11.3)	340,168/902,962 (37.7)	193,073 (17.1)	13,691/192,465 (7.1)	45,341/176,020 (25.8)	1,232,327 (10.9)	130,775/1,227,407 (10.7)	385,509/1,078,982 (35.7)
20–44	5,561,506 (54.9)	564,088/5,538,423 1(0.2)	2,044,632/4,313,280 (47.4)	536,519 (47.5)	38,165/534,790 (7.1)	163,547/481,332 (34.0)	6,098,025 (54.2)	602,253/6,073,213 (9.9)	2,208,179/4,794,612 (46.1)
45–54	1,388,279 (13.7)	151,829/1,382,595 (11.0)	465,781/1,044,003 (44.6)	150,816 (13.3)	11,978/150,192 (8.0)	43,511/130,600 (33.3)	1,539,095 (13.7)	163,807/1,532,787 (10.7)	509,292/1,174,603 (43.4)
55–64	1,240,657 (12.3)	121,718/1,235,830 (9.9)	378,804/933,555 (40.6)	141,644 (12.5)	9,176/141,217 (6.5)	33,959/123,988 (27.4)	1,382,301 (12.3)	130,894/1,377,047 (9.5)	412,763/1,057,543 (39.0)
65–74	614,020 (6.1)	51,364/611,626 (8.4)	160,412/453,740 (35.4)	80,014 (7.1)	3,858/79,756 (4.8)	14,122/69,919 (20.2)	694,034 (6.2)	55,222/691,382 (8.0)	174,534/523,659 (33.3)
75–84	159,570 (1.6)	15,617/158,931 (9.8)	40,304/116,406 (34.6)	23,928 (2.1)	1,114/23,844 (4.7)	3,252/20,861 (15.6)	183,498 (1.6)	16,731/182,775 (9.2)	43,556/137,267 (31.7)
≥85	28,928 (0.3)	3,529/28,789 (12.3)	7,093/21,153 (33.5)	4,564 (0.4)	287/4,544 (6.3)	584/4,029 (14.5)	33,492 (0.3)	3,816/33,333 (11.5)	7,677/25,182 (30.5)
NR	86,926 (0.9)	9,558/85,959 (11.1)	4,519/72,267 (6.3)	193,073 (17.1)	13,691/192,465 (7.1)	0 (—)	96,928 (0.9)	14,266/93,314 (15.3)	4,519/72,267 (6.3)
**Gender**									
Male	4,387,423 (43.3)	488,500/4,368,196 (11.2)	1,463,152/3,463,678 (42.2)	502,376 (44.4)	38,122/500,619 (7.6)	129,437/448,448 (28.9)	4,889,799 (43.4)	526,622/4,868,815 (10.8)	1,592,589/3,912,126 (40.7)
Female	5,553,635 (54.8)	528,193/5,531,368 (9.6)	1,972,138/4,373,187 (45.1)	627,993 (55.6)	40,146/626,001 (6.4)	174,841/558,112 (31.3)	6,181,628 (54.9)	568,339/6,157,369 (9.2)	2,146,979/4,931,299 (43.5)
Other	5,020 (0.1)	318/4,992 (6.4)	1,744/3,130 (55.7)	0 (—)	0 (—)	0 (—)	5,020 (0.0)	318/4,992 (6.4)	1,744/3,130 (55.7)
NR	183,064 (1.8)	22,484/179,894 (12.5)	4,679/17,371 (26.9)	189 (0.0)	1/188 (0.5)	38/189 (20.1)	183,253 (1.6)	22,485/180,082 (12.5)	4,717/17,560 (26.9)

**Abbreviations**: AI/AN = American Indian or Alaska Native; NH/OPI = Native Hawaiian or Other Pacific Islander; NR = not reported.

* Percentage of the total and the number of tested persons is shown.

^†^ Percentage of tests with positive results. The two numbers are the number of tests with positive results and the total number of tested persons with known test results.

^§^ Percentage of tested persons who were symptomatic at testing. The two numbers are the number of persons symptomatic at testing and the total number of tested persons with known symptom status.

^¶^ Race and ethnicity percentages calculated among the total tested population, including those who did not report race or ethnicity. Data reported in the text do not include those who did not report race or ethnicity.

Among symptomatic and asymptomatic community members seeking testing, 17.1% and 5.1%, respectively, received a positive result (Table 2). Among asymptomatic persons, the highest percentages of positive test results were among those aged ≥85 years (7.4%) and <20 years (6.3%) (Table 2). Overall, 82.0% of test results were returned within 2 days (time from sample collection to result reported), with declines to 40.7% in July 2020 and 59.3% in December 2020, corresponding to the first and second peaks in national testing volume and cases (Figure). The percentage of test results returned within 2 days was approximately the same for the Pharmacies+ Testing (82.5%) and Surge Testing (80.7%) programs, though the percentage was lower for Surge Testing through September, 2020. The percentage of CBTS program tests with positive results increased in parallel with increases seen in reported cases nationwide (Supplementary Figure, https://stacks.cdc.gov/view/cdc/111229).

**TABLE 2:** Positive SARS-CoV-2 test result rates by symptom status — Community-Based Testing Sites program, United States, March 2020–September 2021

Characteristic	Positive test results, no./total no. (%)					
	Pharmacies+ Testing sites		Surge Testing sites		Combined sites	
	Symptomatic*	Asymptomatic^†^	Symptomatic*	Asymptomatic^†^	Symptomatic*	Asymptomatic^†^
**Total**	**590,770/3,427,392** **(17.2)**	**239,240/4,399,816** **(5.4)**	**47,069/302,876** **(15.5)**	**20,244/700,340** **(2.9)**	**637,839/3,730,268** **(17.1)**	**259,484/5,100,156** **(5.1)**
**Race, irrespective of ethnicity**						
White	298,851/1,718,578 (17.4)	100,632/2,101,800 (4.8)	31,942/207,761 (15.4)	11,711/437,111 (2.7)	330,793/1,926,339 (17.2)	112,343/2,538,911 (4.4)
AI/AN	4,413/21,730 (20.3)	1,558/20,977 (7.4)	0 (—)	0 (—)	4,413/21,730 (20.3)	1,558/20,977 (7.4)
Asian	24,516/175,116 (14)	10,683/275,136 (3.9)	1,757/15,960 (11.0)	799/67,829 (1.2)	26,273/191,076 (13.8)	11,482/342,965 (3.3)
Black	58,542/347,202 (16.9)	28,935/431,240 (6.7)	3,638/29,724 (12.2)	2,190/76,234 (2.9)	62,180/376,926 (16.5)	31,125/507,474 (6.1)
NH/OPI	2,923/19,738 (14.8)	1,379/37,622 (3.7)	211/2,934 (7.2)	146/16,527 (0.9)	3,134/22,672 (13.8)	1,525/54,149 (2.8)
Other	131,547/794,084 (16.6)	60,672/1,027,219 (5.9)	6,378/29,828 (21.4)	2,737/51,446 (5.3)	137,925/823,912 (16.7)	63,409/1,078,665 (5.9)
NR	69,978/350,944 (19.9)	35,381/505,822 (7.0)	3,143/16,669 (18.9)	2661/51,193 (5.2)	73,121/367,613 (19.9)	38,042/557,015 (6.8)
**Ethnicity, irrespective of race**						
Hispanic	109,464/506,898 (21.6)	49,634/503,179 (9.9)	15,183/68,403 (22.2)	6,659/106,648 (6.2)	124,647/575,301 (21.7)	56,293/609,827 (9.2)
Non-Hispanic	251,337/1,512,103 (16.6)	90,129/1,904,812 (4.73)	21,156/157,890 (13.4)	8,124/377,630 (2.2)	272,493/1,669,993 (16.3)	98,253/2,282,442 (4.3)
NR	229,969/1,408,391 (16.3)	99,477/1,991,825 (5.0)	10,730/76,583 (14.0)	5,461/216,062 (2.5)	240,699/1,484,974 (16.2)	104938/2207887 (4.8)
**Age group, yrs**						
<20	62,339/338,838 (18.4)	38,434/560,867 (6.9)	7,027/45,144 (15.6)	4,867/130,285 (3.7)	69,366/383,982 (18.1)	43,301/691,152 (6.3)
20–44	331,541/2,035,992 (16.3)	113,650/2,260,735 (5.0)	24,502/162,785 (15.1)	8,301/316,899 (2.6)	356,043/2,198,777 (16.2)	121,951/2,577,634 (4.7)
45–54	88,211/463,816 (19.0)	31637/576,214 (5.5)	7,368/43,268 (17.0)	2,793/86,754 (3.2)	95,579/507,084 (18.9)	34,430/662,968 (5.2)
55–64	69,808/377,323 (18.5)	28,141/552,925 (5.1)	5,478/33,817 (16.2)	2,466/89,786 (2.8)	75,286/411,140 (18.3)	30,607/642,711 (4.8)
65–74	28,465/159,780 (17.8)	13,593/292,316 (4.7)	2,041/14,045 (14.5)	1,270/55,637 (2.3)	30,506/173,825 (17.6)	14,863/347,953 (4.3)
75–84	8,138/40,136 (20.3)	4,580/75,830 (6.0)	533/3,236 (16.5)	410/17,550 (2.3)	8,671/43,372 (19.99)	4,990/93,380 (5.3)
≥85	1,706/7,052 (24.2)	1,160/14,002 (8.3)	101/511/512 (20.7)	137/3,429 (4)	1,826/7,633 (23.9)	1,297/17,431 (7.4)
NR	562/4,455 (12.6)	8,045/66,927 (12.0)	0 (—)	4,867/130,285 (3.7)	562/4,455 (12.6)	8,045/66,927 (12.0)
**Gender**						
Male	280,689/1,456,776 (19.3)	117,839/1,992,996 (5.9)	22,711/128,786 (17.6)	10,430/318,005 (3.3)	303,400/1,585,562 (19.1)	128,269/2,311,001 (5.6)
Female	309,428/1,964,274 (15.8)	120,630/2,393,080 (5.0)	24,357/174,053 (14.0)	9,814/382,184 (2.6)	333,785/2,138,327 (15.6)	130,444/2,775,264 (4.7)
Other	191/1,731 (11.03)	66/1,377 (4.79)	0 (—)	0 (—)	191/1,731 (11.03)	66/1,377 (4.79)
NR	462/4,611 (10.02)	705/12,363 (5.7)	1/37 (2.7)	0/151 (0)	463/4,648 (9.96)	705/12,514 (5.63)

**Abbreviations**: AI/AN = American Indian or Alaska Native; NH/OPI = Native Hawaiian or Other Pacific Islander; NR = not reported.

* Positive rate among tested persons who were symptomatic at testing. The two numbers are the number of persons testing positive among those who were symptomatic at testing and the total number of persons who were symptomatic at testing.

^†^ Positive rate among tested persons who were asymptomatic at testing. The two numbers are the number of persons testing positive among those who were asymptomatic at testing and the total number of persons who were asymptomatic at testing.

**FIGURE:**
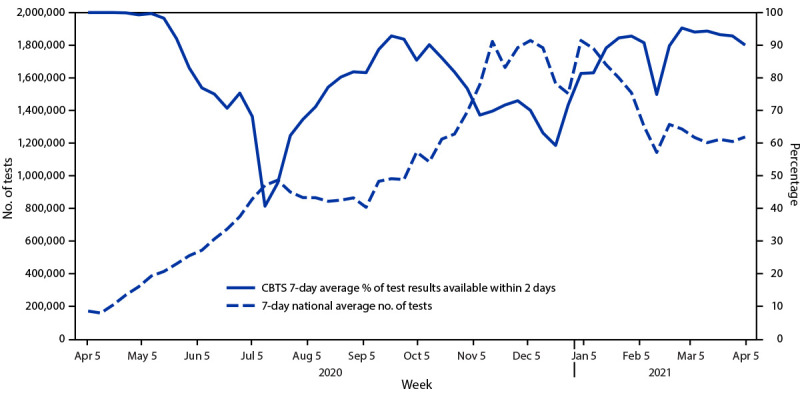
Average number of SARS-CoV-2 tests nationwide and percentage of SARS-CoV-2 tests available within 2 days from the Community-Based Testing Sites Pharmacies+ Testing and Surge Testing programs, by week — United States, April 5, 2020–April 5 2021 **Abbreviation**: CBST = community-based testing sites.

## Discussion

During March 19, 2020–April 11, 2021, the CBTS program conducted 11,661,923 no-charge SARS-CoV-2 tests (approximately 3% of the national testing volume during the same period) at 8,319 locations across the United States and its territories, providing a model for geographically diverse, national, community-centered testing facilities in response to an infectious disease outbreak. Analyses suggest that both symptomatic and asymptomatic persons across a broad distribution of age, race and ethnicity, and sex categories accessed testing through the CBTS program. Results were consistent with other reports showing higher percentages of positive test results among Black, Hispanic, and American Indian or Alaska Native populations (*3*,*4*). Through the combined efforts of federal, state, local, and territorial responders, industry experts, medical suppliers, and service providers, the CBTS program helped meet the diagnostic demands created by an unprecedented public health emergency. Partnerships leveraged across government and the private sector facilitated national reach in a short timeframe.

In April 2021, the CBTS Pharmacies+ Testing and Surge Testing programs were expanded into the ICATT program under the HHS Testing and Diagnostics Work Group (*5*). In the early stages of the pandemic, testing data from CBTS were informative for the tracking of COVID-19 cases and designing continuing response efforts, including the subsequent ICATT program. With funding from the American Rescue Plan, the ICATT program supported school openings and scaled to reach new populations, including testing at crowded public events and for unaccompanied migrating children. As of November 12, 2021, the CBTS and ICATT programs have conducted approximately 26.6 million tests with approximately 10,000 active testing sites. The ICATT program has expanded the reach of its testing through specimen pooling (enhancing efficiency by batching multiple samples for a single test), incentives, mobile pharmacy sites, and point-of-care and self-testing. The program has also contributed to whole genome sequencing of viral isolates and begun linking ICATT program data to self-reported immunization status to identify infections in vaccinated persons. The ICATT program is supported by the HHS Protect platform, integrating approximately 200 separate COVID-19 data sources from federal, state, and local governments, along with data from health care industry partners and nongovernmental organizations.**

Various innovations have been implemented throughout the CBTS program to improve patient safety, conserve testing resources, and expand the program’s reach. For example, a shift from nasopharyngeal swabbing by a medical provider to anterior nares self-swabbing enabled less invasive sample collection, reduced patient-provider contact, conserved personal protective equipment, and eliminated the need for powered air-purifying respirators. Other innovations included the provision of walk-up testing pods in urban areas, video-observed swabbing to reduce patient-provider contact, and mobile teams providing testing at long-term care facilities, essential industry locations, and in underresourced neighborhoods.

The collaborative approach to aligning resources and technical capabilities across partnerships, virtual platforms, and integrated data systems enhanced the success of the CBTS program. Like many SARS-CoV-2 testing operations, the CBTS program experienced periodic, extended turnaround times for receiving results during peak periods of the pandemic (*6*). Delays sometimes extended beyond 10 days, which limits the value of testing in mitigating onward transmission and for supporting persons in their considerations of COVID-19–associated exposure risk (*7*). Considering the high positivity rates among racial and ethnic minorities, use of well constructed vulnerability indices could improve the reach of community-based testing and provide an opportunity to leverage resources in communities most at risk; for example, the Pandemic Vulnerability Index uses county-level data to build local COVID-19 vulnerability measures (*8*).

The findings in this report are subject to at least two limitations. First, persons tested were self-selected from local communities during a period of shifting guidance about who should seek testing; the fact that persons were not randomly selected for testing limits the ability to extrapolate the findings of this report. Finally, age and race and ethnicity data were not collected from all persons being tested, and reasons for test seeking were not ascertained.

This report highlights the value of community-based testing programs in improving access for diagnostic testing, including for symptomatic persons. Lessons learned through administering CBTS and ICATT programs demonstrate the value of cross-sector partnerships and collaboration in aligning resources and technical capabilities for providing testing services that are responsive to local community needs. Efforts should continue to improve the reach of community-based testing in communities most at risk. Although these programs provided a relatively small portion of the overall U.S. SARS-CoV-2 testing needed, their broad geographic reach, successful partnerships, and adaptability serve as a model that can inform current community-based screening, surveillance, and disease control programs and responses to future public health emergencies.

**Summary**

**What is already known about this topic?**

Strong partnerships enable rapid, coordinated responses that support underresourced communities during public health emergencies.

**What is added by this report?**

During March 19, 2020–April 11, 2021, the Community-Based Testing Sites (CBTS) program conducted 11,661,923 SARS-CoV-2 tests at 8,319 locations across the United States and its territories, including 3% administered through Drive-Through Testing, 87% through Pharmacies+ Testing, and 10% through Surge Testing.

**What are the implications for public health practice?**

The CBTS program demonstrated the value of successful partnerships and collaboration for providing testing services that are responsive to local community needs. These lessons can guide current community-based screening, surveillance, and disease control programs and responses to future public health emergencies.

## Footnotes

* Information on race was collected separately from information on ethnicity, and the results for race are reported irrespective of ethnicity and vice versa.

† Sites included counties with higher social vulnerability as measured by the Social Vulnerability Index. Mean Social Vulnerability Index of 0.57, indicating 57% of counties in the nation are less vulnerable than the average of selected sites. https://www.atsdr.cdc.gov/placeandhealth/svi/index.html

§ Decommissioned September 30, 2021.

¶ 45 C.F.R. part 46.102(l)(2); 21 C.F.R. part 56; 42 U.S.C. Sect. 241(d); 5 U.S.C. Sect. 552a; 44 U.S.C. Sect. 3501 et seq.

**
https://protect-public.hhs.gov
